# MRI grading for informed clinical decision-making in Peutz–Jeghers syndrome patients with cervical lesions

**DOI:** 10.1038/s41598-024-75227-1

**Published:** 2024-10-10

**Authors:** Anqi Jiang, Yiqing Chen, Yan Ning, Bing Yu, Hui Wang, Fenghua Ma, Congjian Xu, Yu Kang

**Affiliations:** 1 Obstetrics and Gynecology Hospital of Fudan University, Shanghai, China; 2 Shanghai Key Laboratory of Female Reproductive Endocrine Related Diseases, Shanghai, China; 3 Central Clinical School, The University of Sydney, Sydney, Australia

**Keywords:** Cervical lesions, Gastric-type endocervical adenocarcinoma, Lobular endocervical glandular hyperplasia, Magnetic resonance imaging, Peutz–Jeghers syndrome, Cancer imaging, Reproductive disorders, Cancer

## Abstract

The preoperative diagnosis and management of Peutz–Jeghers syndrome (PJS) patients with cervical lesions remain problematic. This study analysed the associations between pathological types of cervical lesions in PJS patients and their MRI features. A total of 34 PJS patients were included and two experienced radiologists reviewed the MRIs independently. Based on the pathological diagnosis, the patients were categorized into four groups: normal (n  =  4), lobular endocervical glandular hyperplasia (LEGH, n  =  11), atypical LEGH (aLEGH, n  =  8), and gastric-type endocervical adenocarcinoma (G-EAC, n  =  11). By observing the MRI features, we found statistically significant differences in the extent of lesions (P  = 0 .001), distribution of microcysts (P  = 0 .001), proportion of microcysts (P  <  0.001) and endometrial involvement (P  = 0.019) among the four groups. Notably, solid components and disrupted cervical stromal rings were found only in the aLEGH and G-EAC groups (P  < 0.001).  Consequently, we created a novel grading system based on the aforementioned MRI features to align with the potential malignancy of cervical lesions in PJS patients. This system enables patients to receive timely and appropriate treatment recommendations while facilitating collaboration between radiologists and physicians.

**Supplementary Information:**

The online version contains supplementary material available at 10.1038/s41598-024-75227-1.

## Introduction

Peutz–Jeghers syndrome (PJS) is a rare autosomal dominant genetic condition caused by a germline mutation in the STK11 gene. Its clinical manifestations include mucocutaneous pigmentation, Peutz–Jeghers hamartomatous polyps and cancer susceptibility^1^. The gynecological disorders associated with PJS mainly include lobular endocervical glandular hyperplasia (LEGH), gastric-type endocervical adenocarcinoma (G-EAC), and sex cord tumor with annular tubules (SCTAT)^2^.

G-EAC is a special subtype of cervical adenocarcinoma that accounts for 10% of all cervical adenocarcinomas and is the most common subtype unassociated with human papillomavirus (HPV)^3^. Due to the higher lesion location in the endocervical canal, patients are often missed by pelvic examination and Pap tests. Considering the difficulty in detecting G-EAC, it is not difficult to understand why most patients are diagnosed at advanced stages^4^. Another characteristic of G-EAC is propensity to ovarian metastasis, synchronous mucinous metaplasia and neoplasia of the female genital tract. Importantly, G-EAC is a highly invasive tumor with a poor prognosis and limited therapeutic efficacy^5^. Disease-specific survival at 5 years is 42% for G-EAC versus 91% for usual HPV-associated endocervical adenocarcinoma^6^. The lifetime risk of gynecological tumors is significantly increased in women with PJS^7^. Moreover, the incidence of gynecological tumors is high among PJS patients in China and Japan, with G-EAC being the most prevalent. The onset age of precancerous lesions (LEGH and atypical LEGH) is also earlier than that of other lesions^8,9^. However, due to the rarity and complexity of PJS and G-EAC, many challenges currently exist in the diagnosis, management, and follow-up of cervical lesions associated with PJS.

Magnetic resonance imaging (MRI) is an important supplement to ultrasound for the examination of the female reproductive system. MRI can provide high-resolution images of the uterus and ovaries, surpassing ultrasound and computed tomography in diagnosing the origin of pelvic masses. Hence, MRI is considered a useful tool for the diagnosis of cervical multicystic lesions^10^. Additionally, the onset of gynecological diseases in PJS patients tends to be in their childbearing age or even in their teens. MRI has the safety advantage of nonionizing radiation, making it more suitable for follow-up^11^. However, there are no unified criteria for the radiological grade of cervical lesions, and inexperienced radiologists may often misdiagnose or overlook these lesions. However, further research is needed to explore the role of MRI in women with PJS.

Therefore, we conducted a retrospective study on the clinicopathologic and imaging features of 34 patients with PJS. After reviewing the MRIs, we aimed to establish grading criteria to guide clinical decision-making and management in female patients with PJS. Lastly, we applied our findings to 2 newly diagnosed PJS patients who had not undergone biopsy.

## Methods

### Patients and study design

We retrospectively analysed the data of 61 patients with PJS who visited the Obstetrics and Gynecology Hospital of Fudan University from October 2017 to August 2023. The inclusion criteria for patients were as follows: (1) a confirmed diagnosis of PJS by clinicopathologic characteristics and/or genetic test results; (2) a definitive pathological diagnosis of cervical lesions; and (3) clear and complete pelvic MRIs at initial diagnosis. The exclusion criterion was MRIs without adequate quality. Consequently, 34 patients were included in our study. Moreover, we included 2 PJS patients who sought treatment at our hospital in August 2023 without a pathological diagnosis at the time to validate our proposal.

Clinical data included patient age, manifestations, surgical approaches, genetic testing results, and personal and family history. The pathological data included pathological diagnoses. MRIs were obtained with a 1.5T MR scanner (Symphony, Siemens) or 3.0T MR scanner (Ingenia, Philips), with gadopentetate dimeglumine (Magnevist, Bayer Schering Pharma) serving as the contrast agent (0.2 mmol/kg, 2–3 ml/s).

Two radiologists with more than 10 years of experience in abdominal imaging independently reviewed the MRIs. If there were differing opinions, they reached a consensus through consultation. The observed radiological features included the following:

1. The extent of lesions was classified into two types (focal and diffuse) based on the diameter of the lesions being compared with half of the cervical length.
2. The component patterns were classified into cystic, cystic-solid and solid patterns.
3. The size of the cysts was classified into two types (microcyst ≤ 5 mm and macrocyst > 5 mm) based on the diameter.
4. The distribution of microcysts was classified into two types (scattered and dense) based on whether the number of microcysts within the 2 cm × 2 cm observation area exceeded 5.
5. A solid component was defined as a nodular, massive, or infiltrative area shown as a mildly hyperintense area relative to the cervical stroma on T2WI with enhancement on contrast-enhanced T1WI.
6. The intensity of T1WI and T2WI was classified into three types (hypointensity, isointensity and hyperintensity) relative to the outer uterine myometrium. The degree of enhancement was classified as mild, moderate or significant relative to the outer uterine myometrium.
7. Accompanying abnormalities include disrupted cervical stromal ring, ovarian cystic lesions and cervical lesions involving the endometrium.
8. The cervical stromal ring in T2WI refers to the low signal band in the junction area between the mucosa and the epithelial. It is best assessed by evaluating both the sagittal planes that are acquired parallel to the long axis of the cervix.

### Pathologic evaluation

The H&E-stained sections of hysterectomy or conization specimens were evaluated by two pathologists specializing in gynecological pathology and oncology. The diagnosis was made based on the 2020 WHO classification system^12^.

LEGH was characterized by well-formed small- to medium-sized glands with smooth, round outlines containing tall columnar mucinous epithelium with pink-to-clear cytoplasm and bland, basally located nuclei. Atypical LEGH was described as preservation of the prototypical LEGH architecture but with certain atypical features. These included nuclear enlargement, hyperchromasia, distinct nucleoli, and occasional apical mitoses but were not sufficient to classify the condition as carcinoma. G-EAC was defined as an irregularly shaped mucinous gland composed of mucinous cells with granular to foamy cytoplasm. Additionally, tumor clusters displayed eosinophilic to vacuolated cytoplasm with destructive stromal invasion.

### Statistical methods

All the data were statistically analysed using IBM SPSS version 26. Continuous variables are summarized as the mean ± standard deviation, and categorical variables are summarized as the frequency and percentage. Fisher’s exact test was used to assess the associations between imaging features and pathological diagnosis. A value of *p* < 0.05 was considered to indicate statistical significance.

## Results

### Clinicopathologic characteristics

**Fig. 1:**
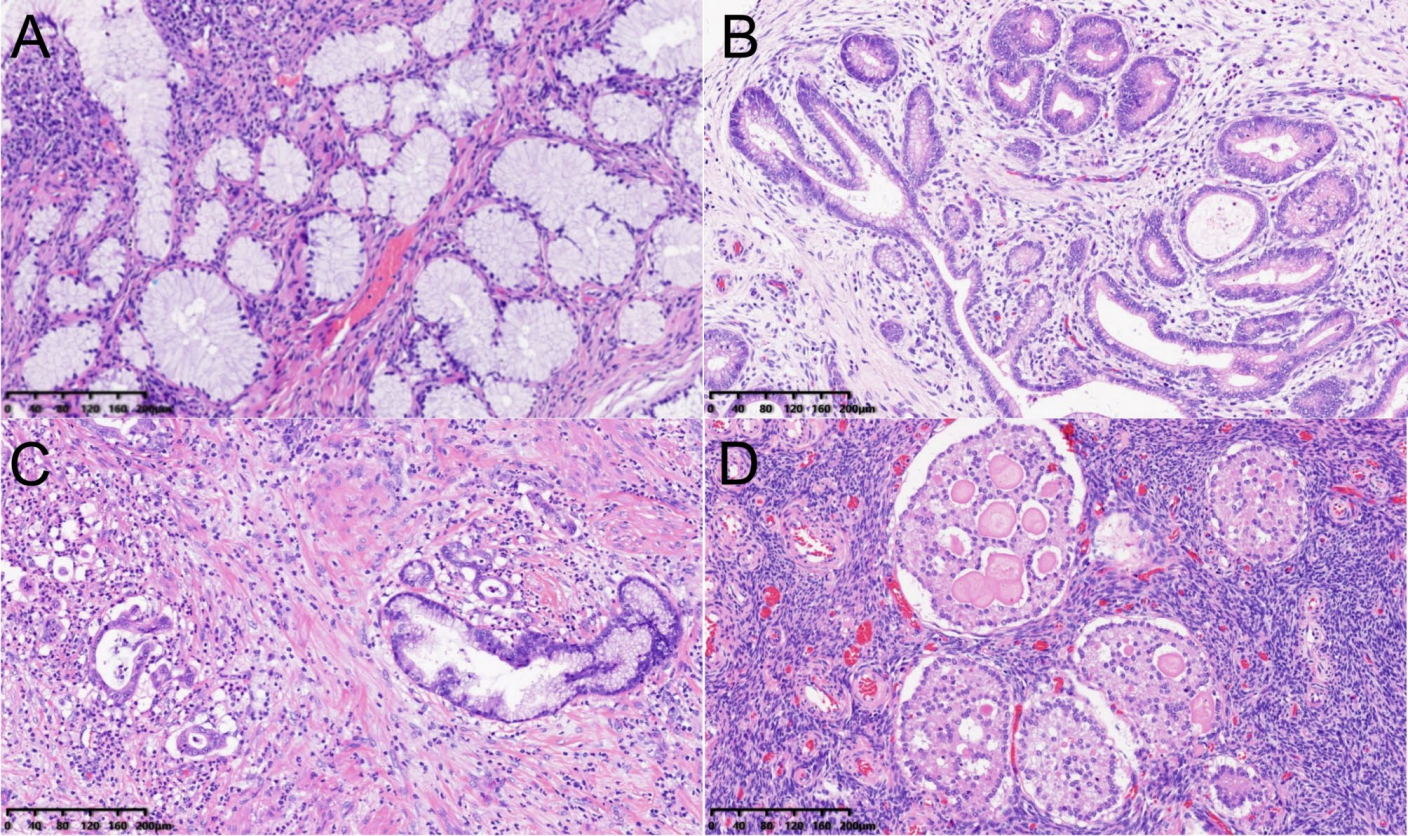
PJS-associated cervical and ovarian lesions (H&E, scale length = 200 μm). Lobular endocervical glandular hyperplasia (LEGH, A): Well-formed small- to medium-sized round glands show smooth round outlines. The glands contained tall columnar mucinous epithelial cells with pink-to-clear cytoplasm and bland, basally located nuclei. Atypical lobular endocervical glandular hyperplasia (aLEGH, B): The preservation of the lobular architecture can be observed. Its characteristics include nuclear enlargement, hyperchromasia, distinct nucleoli and occasional apical mitoses. Gastric-type endocervical adenocarcinoma (G-EAC, C): Patients with irregularly shaped mucinous glands and tumor clusters with destructive stromal invasion. The tumor glands are composed of mucinous cells with granular to foamy cytoplasm, and the tumor clusters exhibit eosinophilic to vacuolated cytoplasm. Sex cord tumor with annular tubules (SCTAT, D): The lesions are characterized by variably sized, rounded nests with simple or complex tubules. Discrete small nests with a conspicuous tubular pattern and typical basement membrane-like material were observed, and the cells were tall and displayed an antipodal distribution within the tubules.

Thirty-four patients included in this study all met the clinical diagnostic criteria for Peutz–Jeghers syndrome. Among them, 26 patients were reported to have a germline variant in STK11 (Table S1). According to the combined pathological and MRI results, 4 (11.8%) patients did not have significant cervical lesions (classified as normal), 11 (32.4%) had LEGH alone, 7 (20.6%) had atypical LEGH (aLEGH) alone, 1 (2.9%) had concurrent aLEGH and gastric-type adenocarcinoma in situ (classified as aLEGH), 7 (20.6%) had G-EAC alone, and 4 (11.8%) had concurrent G-EAC and aLEGH (Fig. 1A–C). The mean age of 34 patients at the time of consultation was 33.2  ±  8.4 (range, 15–62) years. The mean age of the individuals in the precancerous group (n  =  19) was 30.6  ±  7.0 (range, 15–39) years, and that of the individuals in the cancerous group (n  =  11) was 36.7  ±  9.4 (range, 29– 62) years.

A total of 13 patients underwent total or radical hysterectomy with bilateral salpingo-oophorectomy. Among them, 10 had ovarian tumors, including 9 patients with SCTAT (Fig. 1D) (1 patient with concurrent borderline cystadenoma and 1 patient with concurrent adenocarcinoma metastasis), and 1 patient with mucinous cystadenoma.

In terms of clinical manifestations, the most common complication was increased vaginal discharge (20/34, 58.8%). Notably, a significant difference in vaginal discharge was observed among the four groups (*P* = 0.004). All patients had a history of intestinal polyps, intussusception, or perforation. Ten (29.4%) patients were diagnosed with other tumors, including 1 with thyroid cancer combined with concurrent breast cancer, 1 with tubular adenoma, 1 with STK11 adnexal tumor, 1 with intestinal mucinous adenocarcinoma, 1 with breast intraductal papilloma, 1 with breast fibroadenoma, and 4 with ovarian mucinous cystadenoma (1 with concurrent rectal cancer). Regarding family history, 22 (64.7%) had relatives within four generations diagnosed with tumors, and 14 (41.2%) had a family history of PJS (Table 1).

**Table 1:** Clinicopathologic characteristics of patients with peutz–Jeghers syndrome.

Clinicopathologic characteristics		Normal (*n* = 4)	LEGH (*n* = 11)	aLEGH (*n* = 8)	G-EAC (*n* = 11)	*P* value
Mean age (range), y		35.5 (25–44)	29.7 (15–39)	32.0 (29–37)	36.7 (29–62)	0.330
Manifestations	Vaginal discharge	0	5	8	7	0.004
	Vaginal bleeding	0	0	2	2	0.311
Personal history	Intestinal polyps	4	11	8	11	
	Tumors	1	5	1	3	0.452
Family history	Tumors	2	8	6	6	0.675
	PJS	1	7	2	4	0.371
Interventions	Cervical biopsy	4	2	1	9	
	Conization	0	8	6	4	
	Hysteroscopy/D&C	0	8	5	2	
	TH, BSO	0	1	2	0	
	RH, BSO	0	0	0	10	
Accompanying adnexal lesions	SCTAT	N/A	0	1	8	
	Ovarian cystadenoma	N/A	1	0	1	

LEGH, lobular endocervical glandular hyperplasia; aLEGH, atypical lobular endocervical glandular hyperplasia; G-EAC, gastric-type endocervical adenocarcinoma; PJS, Peutz‒Jeghers syndrome; D&C, dilation and curettage; TH, total hysterectomy; BSO, bilateral salpingo-oophorectomy; RH, radical hysterectomy; SCTAT, sex cord tumor with annular tubules; N/A, not applicable.

### MRI features

Although LEGH may present with the specific “cosmos pattern” (dense microcysts in the center of the lesions surrounded by macrocysts)^13^, LEGH, aLEGH and G-EAC often coexist and show overlapping MRI features. When radiologists lack experience or encounter MRIs without characteristic features, they are prone to misdiagnose the lesions as Nabothian cysts or overdiagnose them, which subsequently impacts clinical decision-making^14^. We conducted a comparative analysis between the initial MRI diagnosis at our hospital and the postoperative pathological diagnosis of each patient. The overall consistency rate was 64.7% (22/34), and the consistency rate in the precancerous lesion group was only 57.9% (11/19) (Table S2).

We reviewed the MRIs of each patient to statistically analyse the associations between imaging features and pathological types (Table 2). There were statistically significant differences in the extent of lesions and distribution of microcysts among the four groups (P  = 0.001). Moreover, significant differences in the proportions of microcysts and endometrial involvement were observed among the four groups (P  < 0.001 and P  =  0.019, respectively). Notably, solid components and disrupted cervical stromal rings were observed only in the aLEGH and G-EAC groups (P  <  0.001). The incidence of ovarian cystic lesions did not significantly differ among the four groups (P  = 0.138).

**Table 2:** Imaging features of patients with peutz–Jeghers syndrome.

MRI features		Normal (*n* = 4)	LEGH (*n* = 11)	aLEGH (*n* = 8)	G-EAC (*n* = 11)	*P* value
Extent of lesions^a^	Focal	4 (100%)	2 (18.2%)	1 (12.5%)	0	0.001
	Diffused	0	9 (81.8%)	7 (87.5%)	11 (100%)	
Distribution of microcysts^b^	Scattered	4 (100%)	1 (9.1%)	0	0	0.001
	Dense	0	10 (90.9%)	8 (100%)	7 (63.6%)^c^	
Proportion of microcysts	≥ 0 and ≤ 1/3	4 (100%)	3 (27.3%)	0	8 (72.7%)	< 0.001
	> 1/3 and ≤ 2/3	0	8 (72.7%)	3 (37.5%)	0	
	> 2/3	0	0	5 (62.5%)	3 (27.3%)	
Proportion of solid components	0	4 (100%)	11 (100%)	4 (50%)	0	< 0.001
	≤ 1/2	0	0	4 (50%)	3 (27.3%)	
	> 1/2	0	0	0	8 (72.7%)	
Cervical stromal ring	Intact	4 (100%)	11 (100%)	7 (87.5%)	1 (9.1%)	< 0.001
	Incomplete	0	0	1 (12.5%)	7 (63.6%)	
	Missing	0	0	0	3 (27.3%)	
Ovarian cystic lesions		0	3 (27.3%)	3 (37.5%)	7 (63.6%)	0.138
Endometrium involvement		0	2 (18.2%)	4 (50%)	8 (72.7%)	0.019

LEGH, lobular endocervical glandular hyperplasia; aLEGH, atypical lobular endocervical glandular hyperplasia; G-EAC, gastric-type endocervical adenocarcinoma.

^a^We defined “focal” as a lesion diameter less than half of the cervical length, otherwise “diffused”; ^b^we defined “scattered” as a lesion with fewer than five microcysts within any 2 cm×2 cm observation area, otherwise “dense”; ^c^ we excluded 4 G-EAC patients whose lesions were all solid components.

### MRI grading criteria to guide clinical decision-making

Based on the analysis of the associations between MRI features and pathological types, we proposed novel criteria for MRI grading of cervical lesions in PJS patients (Table 3). We observed highly significant differences (p  <  0.001) in the presence of solid components and the proportion of microcysts between the malignant and non-malignant lesion groups.

**Table 3:** Grading criteria.

Grade (Diagnosis)	MRI features
1 (N)	No obvious lesions were observed.
2 (LB)	1. The proportion of microcysts was > 0 but ≤ 1/3.
	2. No solid components were observed.
3 (US)	1. The proportion of microcysts was > 1/3 and ≤ 2/3.
	2. No solid components were observed.
4 (LM)	1. The proportion of microcysts was > 1/3.
	2. Solid components may be observed but ≤ 1/2.
	3. Cervical stromal ring may be incomplete.
	4. Endometrium involvement may be observed.
5 (M)	1. Solid components were observed and > 1/2.
	2. Cervical stromal ring was incomplete or missing.
	3. Endometrium involvement may be observed.

N, negative; LB, likely benign; US, uncertain significance; LM, likely malignant; HM, highly malignant.

**Fig. 2:**
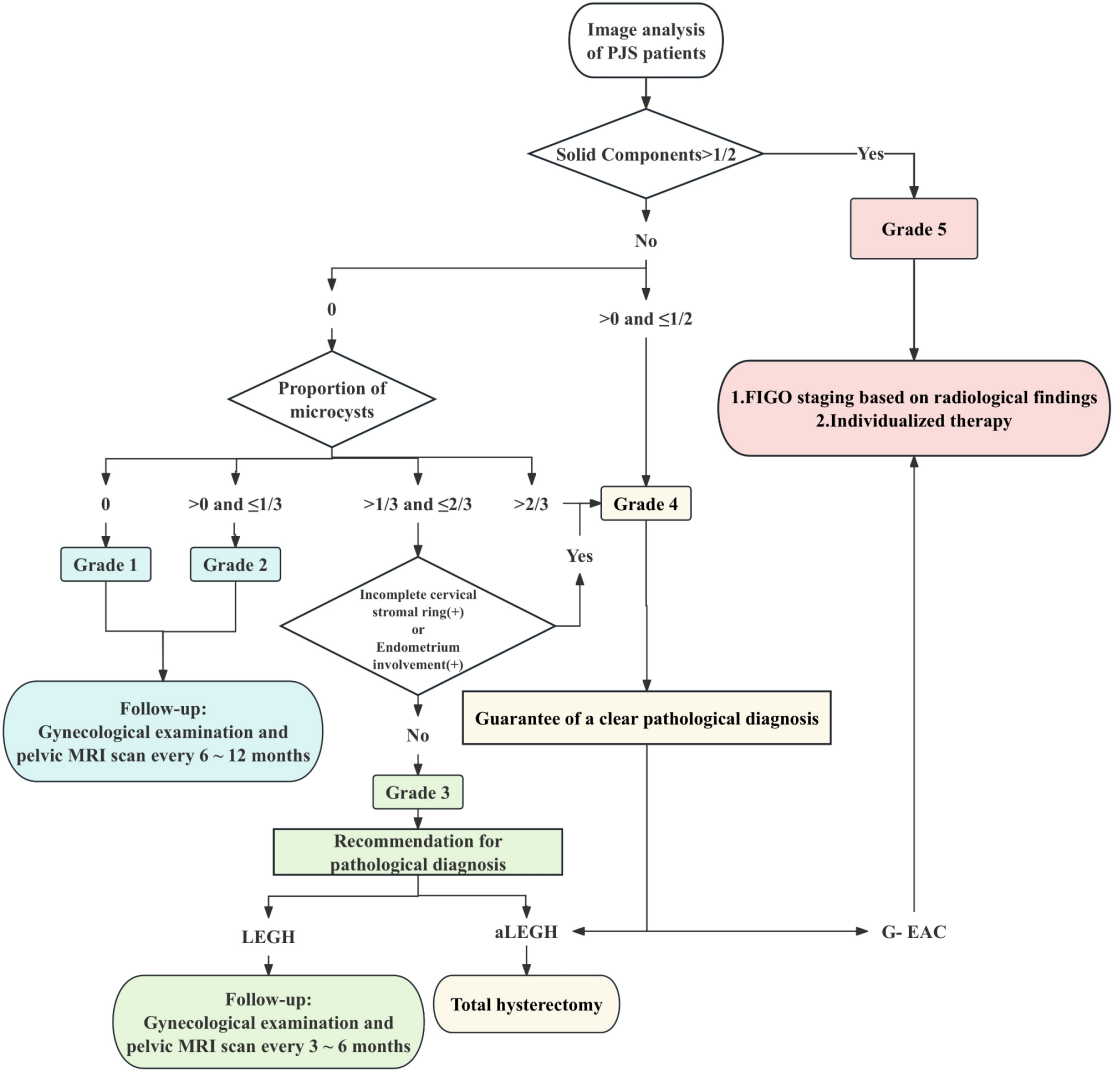
Flowchart of MRI grading for clinical decision-making.

**Fig. 3:**
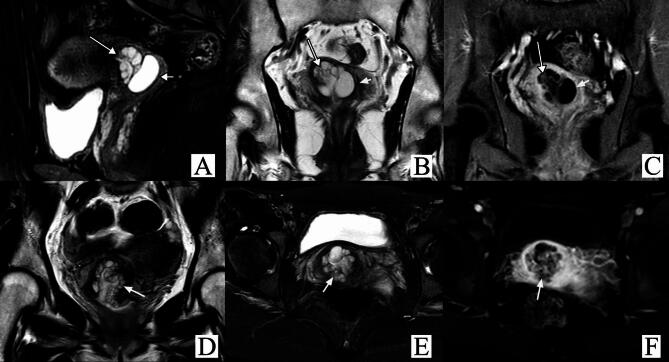
MRI of 2 PJS patients with LEGH. A–C: A 38-year-old patient was classified as Grade 2. She was pathologically confirmed to be LEGH. Sagittal (A) and coronal (B) T2WI showed dense microcysts (long arrow) and macrocysts (short arrow) in the cervical canal. The cervical stromal ring was intact with a low signal density on T2WI (B), and the proportion of microcysts was less than 1/3. Coronal contrast-enhanced T1WI (C) showed that the microcysts were mildly enhanced. D–F: A 27-year-old patient was classified as Grade 3. She was pathologically confirmed to have LEGH with increased vaginal discharge for more than 1 year. Coronal (D) and axial (E) T2WI showed dense microcysts and macrocysts in the cervical canal (arrow). The cervical stromal ring was intact with a low signal density on T2WI (E), and the proportion of microcysts was more than 1/3 but less than 2/3. Axial contrast-enhanced T1WI (F) showed that the microcysts were moderately enhanced.

**Fig. 4:**
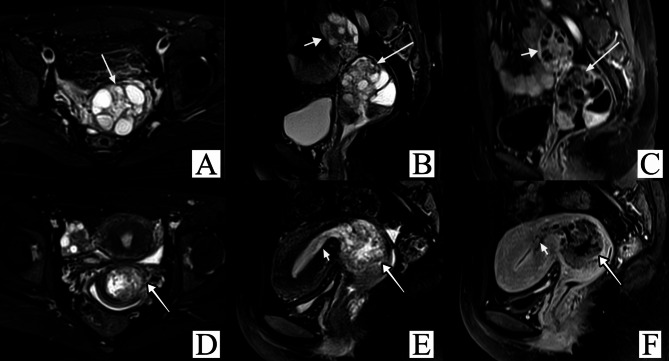
MRI of 2 PJS patients with G-EAC. A-C: A 31-year-old patient was classified as Grade 4. She was pathologically confirmed to have G-EAC concurrent with aLEGH and SCTAT with increased vaginal discharge for more than 10 years. Axial (A) and sagittal (B) T2WI showed dense microcysts surrounded by macrocysts (long arrow). Multiple small cysts could be observed in the left ovary (short arrow), and the cervical stromal ring was incomplete (B). The proportion of solid components was visible and less than 1/2. Sagittal contrast-enhanced T1WI (C) showed that the microcysts were moderately enhanced. D–F: A 31-year-old patient was classified as Grade 5. She was pathologically confirmed to have G-EAC concurrent with SCTAT. Axial (D) and sagittal (E) T2WI showed a diffuse and predominantly solid mass (long arrow) throughout the entire cervix. Local extension of lesions into the endometrium can be observed on sagittal T2WI (short arrow), and the cervical stromal ring was incomplete (E). Sagittal contrast-enhanced T1WI (F) showed that the proportion of solid components was more than 1/2 and was mildly enhanced.

Therefore, we constructed a flowchart for the purpose of helping both radiologists and clinical physicians make informed decisions (Fig. 2). We applied the grading criteria to rediagnose the images of 34 PJS patients. Consequently, only 2 patients with LEGH were overdiagnosed and classified as Grade 4, for an overall consistency rate of 94.1% (32/34) and a consistency rate of 89.5% (17/19) in the precancerous lesion group (Table 4; Figs. 3 and 4).

**Table 4:** Imaging rediagnosis using the grading criteria.

Grade (Diagnosis)	Normal (*n* = 4)	LEGH (*n* = 11)	aLEGH (*n* = 8)	G-EAC (*n* = 11)
1 (N)	4 (100%)	0	0	0
2 (LB)	0	3 (27.3%)	0	0
3 (US)	0	6 (54.5%)	2 (25%)	0
4 (LM)	0	2 (18.2%)	6 (75%)	3 (27.3%)
5 (HM)	0	0	0	8 (72.7%)
Consistency rate	100%	81.8%	100%	100%

LEGH, lobular endocervical glandular hyperplasia; aLEGH, atypical lobular endocervical glandular hyperplasia; G-EAC, gastric-type endocervical adenocarcinoma; N, negative; LB, likely benign; US, uncertain significance; LM, likely malignant; HM, highly malignant.

While certain studies have indicated a limited occurrence of malignant transformation in LEGH^10,13^, it is crucial to acknowledge the elevated risk and poor prognosis of G-EAC in PJS patients^4–9,15,16^. Therefore, we recommend that individuals classified as Grade 1 or 2 opt for imaging surveillance at intervals of every 6–12 months for follow-up. For Grade 3 patients, for whom imaging is insufficient to determine the nature of the lesions, we recommend obtaining a precise pathological diagnosis for more frequent follow-up or subsequent intervention. When imaging strongly indicates the presence of malignant lesions, diagnostic surgery is still the cornerstone of treatment for Grade 4 or 5 patients. For patients with G-EAC, individualized treatment should be based on radiological findings for FIGO staging.

Moreover, we applied our grading criteria to two PJS patients who did not have a pathological diagnosis at the time (Table S1: case 35 and 36). Both were classified as Grade 3 after image analysis, so we recommended surgery to clarify the pathological diagnosis (Fig. S1). After conization, one patient was pathologically diagnosed with LEGH, and the other was diagnosed with aLEGH. We subsequently recommended that the former patient undergo follow-up examinations every 3–6 months, while the latter underwent total hysterectomy.

## Discussion

In recent years, many studies have attempted to differentiate LEGH and G-EAC from clinical and radiological perspectives. Omori et al.^17^ divided cervical multicystic lesions on MRIs into two types and reported that the raspberry type, which has many tiny, closely aggregated cysts, indicated adenocarcinoma in situ in postmenopausal women. Yoshino et al.^14^ reported that the most common manifestation was watery discharge (27.4%) in patients with cervical proliferative glandular lesions. MRI was also used to classify malignant potential into four types according to the location of the lesion and the component patterns. Like in previous studies, we agreed that solid components may indicate malignancy and that the “cosmos pattern” may indicate LEGH^13,17^. Nevertheless, we also observed solid components in 4 aLEGH patients, suggesting that these lesions do overlap in MRI features and should be taken seriously.

However, a pressing clinical focus in PJS is the management of patients with multicystic lesions without solid components^18^. Compared with these studies, we not only observed the component patterns but also further assessed the proportion of microcystic and solid components. Moreover, the disrupted cervical stromal ring and endometrial involvement suggested increased malignant potential^19–22^. Overall, our work and these publications were dedicated to improving the accuracy of the preoperative diagnosis of cervical multicystic lesions.

A poor prognosis has been reported for G-EAC. In this study, 3 patients with G-EAC were lost to follow-up. Among the remaining 8 patients followed up for one year, 2 died (at 8 and 9 months post-operation, respectively), while 6 showed no recurrence or metastasis. Two patients with a poor prognoses tended to be rated as Grade 5 and have solid components > 1/2, missing cervical stormal ring, high stages (IV A), and associated ovarian lesions. In comparison to the favorable prognosis reported by Park et al.^23^, we presume that the higher recurrence and death rates may be attributed to the fact that all patients in our cohort have PJS and carry STK11 germline mutations^4–9,15^. Subsequently, increasing the follow-up duration and enlarging our patient cohort will enable us to draw more compelling conclusions.

The National Comprehensive Cancer Network (NCCN) offers recommendations for monitoring individuals diagnosed with Peutz–Jeghers syndrome. To mitigate the potential risk of gynecologic malignancies, especially G-EAC, the NCCN recommends annual pelvic examinations and Pap tests starting at 18–20 years of age and considering total hysterectomy (including uterus and cervix) once completed with childbearing^16^. Based on the NCCN guidelines, our study developed more specific and targeted management recommendations by grading pelvic MRI in patients with PJS. We aimed to conduct follow-up and monitoring for patients who had undergone the least invasive methods.

The large sample size was one of our strengths. In the context of Peutz–Jeghers syndrome, there were a total of 61 patients treated at Obstetrics and Gynecology Hospital of Fudan University, 34 of whom met the inclusion criteria. Enrollment criteria included comprehensive clinical, pathological, and imaging data, ensuring a well-documented cohort. In addition, the consistency rate of the preoperative diagnosis of cervical multicystic lesions improved when using our diagnostic criteria. Importantly, our grading criteria will serve as a connecting bridge between radiologists and clinical physicians.

There are several limitations to our study. First, we retrospectively analysed only those patients who had a pathological diagnosis, which may have caused selection bias. Most likely, due to selection bias, we did not observe a significant difference in age between groups with different pathological types (P  = 0.330). Second, we lacked observational studies of dynamic changes in imaging features. Kobara et al.^13^ reported that an increase in lesion size of more than 38% may be closely related to the onset of malignant transformation. We may compare follow-up MRIs in the future. Finally, preoperative malignancy grading of G-EAC in patients with PJS was performed mainly by MRI features. In consideration of these limitations, we will refine our multimodal scoring in subsequent studies by incorporating factors such as age, STK11 mutation type, personal and family history, among others. This personalized approach is crucial for genetic counseling and clinical decision-making. Furthermore, we will expand the PJS cohort and prolong follow-up periods in prospective studies to bolster the credibility of our findings. While we acknowledge these limitations and biases, we believe our present work is meaningful for the diagnosis and personalized management of Peutz–Jeghers syndrome.

## Conclusions

We proposed practical MRI grading criteria for Peutz–Jeghers syndrome patients, a high-risk group for gastric-type endocervical adenocarcinoma. The primary assessments in these criteria are the proportion of solid components and microcysts, with additional attention given to the integrity of the cervical stromal ring and endometrial involvement. Building upon these grading criteria, we further propose specific clinical decision-making recommendations for PJS patients, especially those in precancerous conditions. We advocate for using periodic MRI scans as an alternative to invasive examinations for patients with a low likelihood of malignancy, whereas we still recommend definitive pathological diagnosis for those with a high likelihood of malignancy.

## Electronic supplementary material

Below is the link to the electronic supplementary material.

Supplementary Material 1

Supplementary Material 2

Supplementary Material 3

Supplementary Material 4

## Data Availability

No datasets were generated or analysed during the current study.

## Abbreviations

aLEGH: Atypical lobular endocervical glandular hyperplasia
G- EAC: Gastric-type endocervical adenocarcinoma
HPV: Human papillomavirus
LEGH: Lobular endocervical glandular hyperplasia
MRI: Magnetic resonance imaging
NCCN: National Comprehensive Cancer Network
PJS: Peutz–Jeghers syndrome
SCTAT: Sex cord tumor with annular tubules
STK11: Serine/threonine kinase 11
