# Supplementary material for: MRI grading for informed clinical decision-making in Peutz–Jeghers syndrome patients with cervical lesions
Source: Sci Rep. 2024 Oct 10;14:23731. doi: 10.1038/s41598-024-75227-1 (PMC11467353; doi:10.1038/s41598-024-75227-1)
Supplement: Supplementary file 1 — Supplementary Material 1 [file 41598_2024_75227_MOESM1_ESM.docx]

**Table S1 Summary of 36 patients with Peutz-Jeghers syndrome**

| Case | Age,y | STK11 cDNA change | Symptoms | Image features | MRI Grade | Interventions | Pathological diagnosis | FIGO staging |
| --- | --- | --- | --- | --- | --- | --- | --- | --- |
| 1 | 44 | c.866T>G | N/A | No obvious lesions were observed | 1 | Cervical biopsy | Normal |  |
| 2 | 33 | c.837dupC | N/A | No obvious lesions were observed | 1 | Cervical biopsy | Normal |  |
| 3 | 40 | c.402_403dup | N/A | No obvious lesions were observed | 1 | Cervical biopsy | Normal |  |
| 4 | 25 | c.91del | N/A | No obvious lesions were observed | 1 | Cervical biopsy | Normal |  |
| 5 | 28 | c.790_793del | Vaginal discharge | Focal lesions;  Dense microcysts > 1/3 and ≤ 2/3 | 3 | Conization | LEGH |  |
| 6 | 15 | exon1-3del | N/A | Diffused lesions;  Dense microcysts > 1/3 and ≤ 2/3 | 3 | Cervical biopsy | LEGH |  |
| 7 | 19 | exon2-6del | Vaginal discharge | Diffused lesions;  Dense microcysts > 1/3 and ≤ 2/3 | 3 | Cervical biopsy | LEGH |  |
| 8 | 36 | N/A | N/A | Focal lesions;  Dense microcysts > 0 and ≤ 1/3 | 2 | Conization, Hysteroscopy | LEGH |  |
| 9 | 21 | c.137_138del | N/A | Diffused lesions;  Dense microcysts > 1/3 and ≤ 2/3；  Ovarian cystic lesions;  Endometrium involvement | 4 | Conization, Hysteroscopy | LEGH |  |
| 10 | 39 | c.417dupG | N/A | Diffused lesions;  Scattered microcysts > 0 and ≤ 1/3 | 2 | Conization, Hysteroscopy | LEGH |  |
| 11 | 28 | c.890G>A | N/A | Diffused lesions;  Dense microcysts > 1/3 and ≤ 2/3 | 3 | Hysteroscopy | LEGH |  |
| 12 | 38 | c.644del | Vaginal discharge | Diffused lesions;  Dense microcysts > 0 and ≤ 1/3 | 2 | Conization, D&C | LEGH |  |
| 13 | 27 | c.734+1G>A | Vaginal discharge | Diffused lesions;  Dense microcysts > 1/3 and ≤ 2/3；  Endometrium involvement | 4 | Conization, Hysteroscopy | LEGH |  |
| 14 | 38 | c.471del | N/A | Diffused lesions;  Dense microcysts > 1/3 and ≤ 2/3;  Ovarian cystic lesions | 3 | Conization, Hysteroscopy | LEGH |  |
| 15 | 38 | c.863-8_870del | Vaginal discharge | Diffused lesions;  Dense microcysts > 1/3 and ≤ 2/3;  Ovarian cystic lesions | 3 | Conization, Hysteroscopy, TH, BSO | LEGH;  mucinous cystadenoma |  |
| 16 | 28 | N/A | Vaginal discharge | Diffused lesions;  Dense microcysts > 2/3;  Ovarian cystic lesions | 4 | Conization, Hysteroscopy | aLEGH |  |
| 17 | 29 | N/A | Vaginal discharge | Diffused lesions;  Dense microcysts > 1/3 and ≤ 2/3 | 3 | Conization | aLEGH |  |
| 18 | 34 | c.471del | Vaginal discharge | Diffused lesions;  Solid components > 0 and ≤ 1/2;  Dense microcysts > 2/3;  Incomplete cervical stromal ring | 4 | Conization, Hysteroscopy | aLEGH |  |
| 19 | 30 | c.922T>C | Vaginal discharge | Diffused lesions;  Dense microcysts > 1/3 and ≤ 2/3 | 3 | Conization, Hysteroscopy | aLEGH |  |
| 20 | 28 | exon2-3del | Vaginal discharge | Diffused lesions;  Dense microcysts > 1/3 and ≤ 2/3;  Endometrium involvement | 4 | Conization, Hysteroscopy | aLEGH |  |
| 21 | 37 | N/A | Vaginal discharge;  Vaginal bleeding | Diffused lesions;  Solid components > 0 and ≤ 1/2;  Dense microcysts > 2/3;  Ovarian cystic lesions；  Endometrium involvement | 4 | Hysteroscopy,  TH, BSO | aLEGH |  |
| 22 | 38 | c.298C>T | Vaginal discharge | Diffused lesions;  Solid components > 0 and ≤ 1/2;  Dense microcysts > 2/3;  Ovarian cystic lesions；  Endometrium involvement | 4 | Conization, TH, BSO | AIS, aLEGH;  SCTAT |  |
| 23 | 33 | c.580G>A | Vaginal discharge;  Vaginal bleeding | Diffused lesions;  Solid components > 0 and ≤ 1/2;  Dense microcysts > 2/3;  Endometrium involvement | 4 | Cervical biopsy | aLEGH |  |
| 24 | 38 | N/A | N/A | Diffused lesions;  Solid pattern;  Missing cervical stromal ring；  Ovarian cystic lesions；  Endometrium involvement | 5 | Cervical biopsy | G-EAC | IV A |
| 25 | 29 | c.921-1G>C | Vaginal discharge | Diffused lesions; Solid pattern;  Incomplete cervical stromal ring；  Ovarian cystic lesions；  Endometrium involvement | 5 | Cervical biopsy, RH, BSO | 1. EAC;   SCTAT | III C2 |
| 26 | 31 | N/A | N/A | Diffused lesions;  Solid components > 1/2;  Dense microcysts > 0 and ≤ 1/3;  Incomplete cervical stromal ring；  Endometrium involvement | 5 | Cervical biopsy, RH, BSO | G-EAC;  SCTAT | III C1 |
| 27 | 30 | N/A | Vaginal bleeding | Diffused lesions;  Solid pattern;  Missing cervical stromal ring；  Ovarian cystic lesions；  Endometrium involvement | 5 | Cervical biopsy, RH, BSO | G-EAC;  SCTAT, adenocarcinoma metastasis | IV A |
| 28 | 29 | c.250A>T | Vaginal discharge | Diffused lesions;  Solid components > 0 and ≤ 1/2;  Dense microcysts > 2/3;  Incomplete cervical stromal ring;  Ovarian cystic lesions;  Endometrium involvement | 4 | Cervical biopsy, Conization,  D&C, RH, BSO | G-EAC, aLEGH;  SCTAT | I A1 |
| 29 | 41 | c.598_602del | Vaginal discharge | Diffused lesions;  Solid components > 1/2;  Dense microcysts > 0 and ≤ 1/3;  Incomplete cervical stromal ring；  Endometrium involvement | 5 | Cervical biopsy,  RH, BSO | G-EAC;  SCTAT | III C1 |
| 30 | 32 | c.C661T  c.G667T | Vaginal discharge | Diffused lesions;  Solid components > 1/2;  Dense microcysts > 0 and ≤ 1/3;  Incomplete cervical stromal ring | 5 | Cervical biopsy,  RH, BSO | G-EAC;  SCTAT | III C1 |
| 31 | 37 | c.290+1G>A | Vaginal discharge | Diffused lesions;  Solid components > 0 and ≤ 1/2;  Dense microcysts > 2/3;  Ovarian cystic lesions | 4 | Conization,  Hysteroscopy, RH, BSO | G-EAC, aLEGH | I B1 |
| 32 | 31 | c.716G>C | Vaginal discharge | Diffused lesions;  Solid components > 0 and ≤ 1/2;  Dense microcysts > 2/3;  Incomplete cervical stromal ring;  Ovarian cystic lesions | 4 | Conization, RH, BSO | G-EAC, aLEGH;  SCTAT | I A1 |
| 33 | 62 | N/A | Vaginal bleeding | Diffused lesions;  Solid pattern;  Missing cervical stromal ring;  Endometrium involvement | 5 | Cervical biopsy,  RH, BSO | G-EAC | II A2 |
| 34 | 44 | c.T551C | Vaginal discharge | Diffused lesions;  Solid components > 1/2;  Dense microcysts > 0 and ≤ 1/3;  Incomplete cervical stromal ring;  Ovarian cystic lesions;  Endometrium involvement | 5 | Cervical biopsy,  Conization,  RH, BSO | G-EAC, aLEGH;  SCTAT, borderline cystadenoma | I A2 |
| 35 | 34 | N/A | Vaginal discharge | Diffused lesions;  Dense microcysts > 1/3 and ≤ 2/3 | 3 | Conization, RH, BSO | aLEGH |  |
| 36 | 34 | N/A | Vaginal discharge | Diffused lesions;  Dense microcysts > 1/3 and ≤ 2/3；  Ovarian cystic lesions | 3 | Conization, D&C | LEGH |  |

STK11, serine/threonine kinase 11; cDNA, complementary deoxyribonucleic acid; MRI, magnetic resonance imaging; FIGO, International Federation of Gynecology and Obstetrics; N/A, not applicable; LEGH, lobular endocervical glandular hyperplasia; aLEGH, atypical lobular endocervical glandular hyperplasia; AIS, adenocarcinoma in situ; G-EAC, gastric-type endocervical adenocarcinoma; D&C, dilation and curettage; TH, total hysterectomy; BSO, bilateral salpingo-oophorectomy; RH, radical hysterectomy; SCTAT, sex cord tumor with annular tubules.
