# Supplementary material for: MRI grading for informed clinical decision-making in Peutz–Jeghers syndrome patients with cervical lesions
Source: Sci Rep. 2024 Oct 10;14:23731. doi: 10.1038/s41598-024-75227-1 (PMC11467353; doi:10.1038/s41598-024-75227-1)
Supplement: Supplementary file 2 — Supplementary Material 2 [file 41598_2024_75227_MOESM2_ESM.docx]

**Table S2 Accuracy rate of initial imaging diagnosis**

| Initial imaging diagnosis | Normal  (n=4) | LEGH+aLEGH  (n=19) | G-EAC  (n=11) |
| --- | --- | --- | --- |
| Normal | 3 (75%) | 0 | 0 |
| LEGH | 1 (25%) | 11 (57.9%) | 1 (9.1%) |
| G-EAC | 0 | 3 (15.8%) | 8 (72.7%) |
| Nonspecific | 0 | 5 (21.1%) | 2 (18.2%) |
| Consistency rate | 75% | 57.9% | 72.7% |

LEGH, lobular endocervical glandular hyperplasia; aLEGH, atypical lobular endocervical glandular hyperplasia; G-EAC, gastric-type endocervical adenocarcinoma.
