# Supplementary figures and images for: MRI grading for informed clinical decision-making in Peutz–Jeghers syndrome patients with cervical lesions
Source: Sci Rep. 2024 Oct 10;14:23731. doi: 10.1038/s41598-024-75227-1 (PMC11467353; doi:10.1038/s41598-024-75227-1)

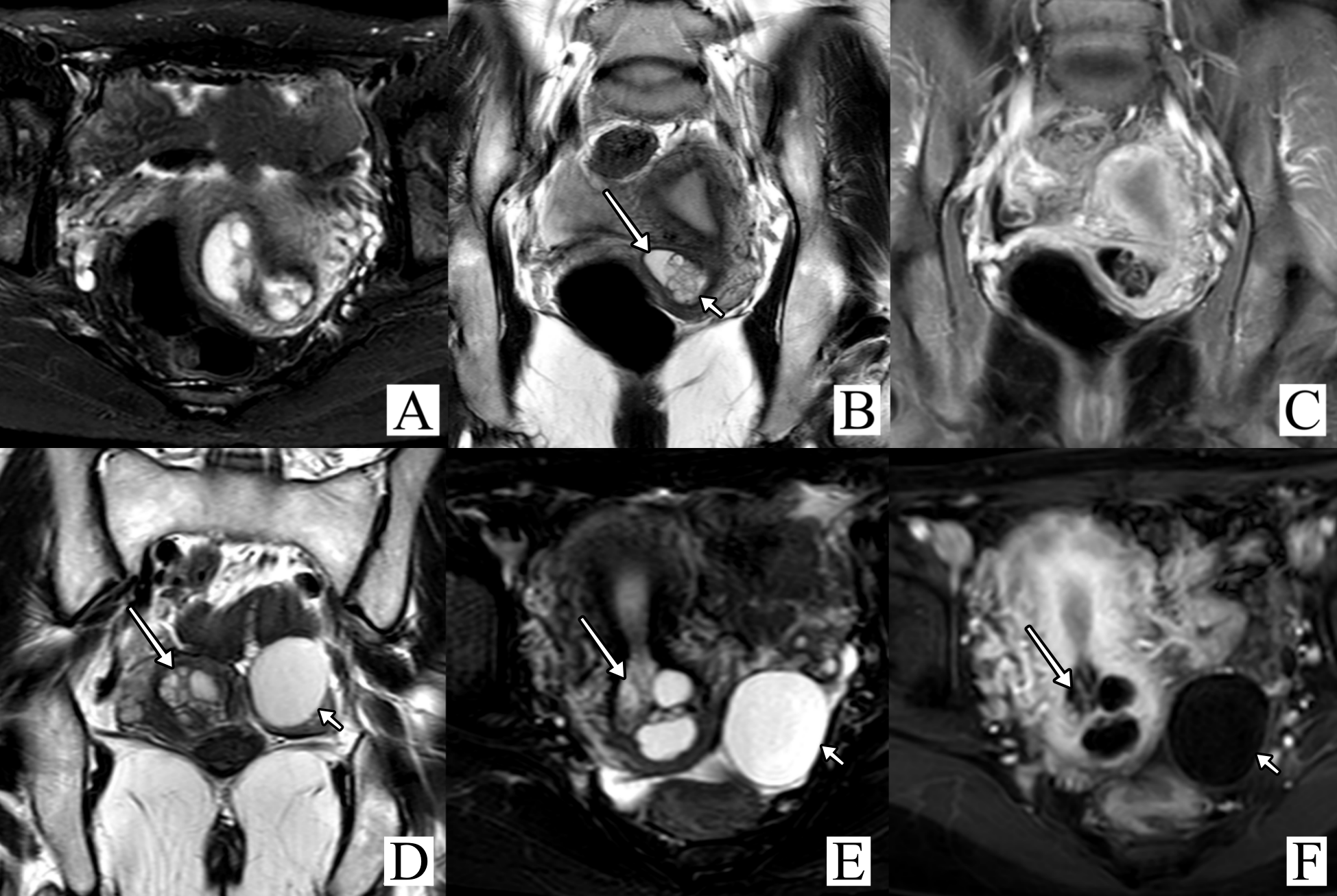

Supplement: Supplementary file 3 — Supplementary Material 3 [file 41598_2024_75227_MOESM3_ESM.tiff]
