# Supplementary material for: MRI grading for informed clinical decision-making in Peutz–Jeghers syndrome patients with cervical lesions
Source: Sci Rep. 2024 Oct 10;14:23731. doi: 10.1038/s41598-024-75227-1 (PMC11467353; doi:10.1038/s41598-024-75227-1)
Supplement: Supplementary file 4 — Supplementary Material 4 [file 41598_2024_75227_MOESM4_ESM.docx]

**Fig. S1** MRI of 2 PJS patients classified as Grade 3 preoperatively.

A-C: A 34-year-old woman with increased vaginal discharge for more than 1 year was classified as Grade 3 and then was pathologically confirmed as aLEGH. Axial (A) and coronal (B) T2WI showed dense microcysts (short arrow) and macrocysts (long arrow) in the middle and upper sections of the cervical canal. The cervical stromal ring was intact with low signal on T2WI (B) and the proportion of microcysts was more than 1/3 but less than 2/3. Coronal contrast-enhanced T1WI (C) showed the microcysts were moderately enhanced.

D-F: A 34-year-old woman with increased vaginal discharge for more than 4 years was classified as Grade 3 and then was pathologically confirmed as LEGH. Coronal (D) and axial (E) T2WI showed dense microcysts and macrocysts (long arrow) in the middle and upper sections of the cervical canal. The cervical stromal ring was intact with low signal on T2WI (E) and the proportion of microcysts was more than 1/3 but less than 2/3. A pure cyst with a diameter of 3 cm was observed in the left ovary (short arrow). Axial contrast-enhanced T1WI (F) showed the microcysts were moderately enhanced.
